# Soft back exosuit controlled by neuro-mechanical modeling provides adaptive assistance while lifting unknown loads and reduces lumbosacral compression forces

**DOI:** 10.1017/wtc.2025.3

**Published:** 2025-02-24

**Authors:** Alejandro Moya-Esteban, Mohamed Irfan Refai, Saivimal Sridar, Herman van der Kooij, Massimo Sartori

**Affiliations:** Department of Biomechanical Engineering, University of Twente, Enschede, The Netherlands

**Keywords:** soft wearable robotics, neuro-robotics, biomechatronics, human motor control, exosuits

## Abstract

State-of-the-art controllers for active back exosuits rely on body kinematics and state machines. These controllers do not continuously target the lumbosacral compression forces or adapt to unknown external loads. The use of additional contact or load detection could make such controllers more adaptive; however, it can be impractical for daily use. Here, we developed a novel neuro-mechanical model-based controller (NMBC) that uses a personalized electromyography (EMG)-driven musculoskeletal (MSK) model to estimate lumbosacral joint loading. NMBC provided adaptive, subject- and load-specific assistive forces proportional to estimates of the active part of biological joint moments through a soft back support exosuit. Without *a priori* information, the maximum assistive forces of the cable were modulated across weights. Simultaneously, we applied a non-adaptive, kinematic-dependent, trunk inclination-based controller (TIBC). Both NMBC and TIBC reduced the mean and peak biomechanical metrics, although not all reductions were significant. TIBC did not modulate assistance across weights. NMBC showed larger reductions of mean than peak values, significant reductions during the erect stance and the cumulative compressive loads by 21% over multiple cycles in a cohort of 10 participants. Overall, NMBC targeted mean lumbosacral compressive forces during lifting without *a priori* information of the load being carried. This may facilitate the adoption of non-hindering wearable robotics in real-life scenarios. As NMBC is informed by an EMG-driven MSK model, it is possible to tune the timing of NMBC-generated torque commands to the exosuit (delaying or anticipating commands with respect to biological torques) to target further reduction of peak or mean compressive forces and muscle fatigue.

## 1. Introduction

Low back pain (LBP) is a worldwide clinical, economical, and public health problem, owing to its effects on daily life activity limitation and work absence. About 75%–85% of the population will experience a form of LBP at some stage of their life (Andersson, 1998). For workers in occupational environments, such as factories and hospitals, repetitive handling of heavy loads and non-ergonomic postures constitutes a main risk for LBP development. Repetitive and heavy material handling entails high cumulative compression and shear intervertebral forces in the human spine and may contribute to LBP development, due to prolapse or protrusion of spinal intervertebral disks or damage to vertebral joints (Brinckmann et al., 1988; Norman et al., 1998).

Within occupational environments, several measures have been introduced to minimize the incidence of LBP, including training workers on correct ergonomic postures, adapting workspaces to facilitate ergonomic postures (Rodrigues and Rocha, 2022), introducing weight limits (Waters et al., 1993), or using external manipulators, such as cranes, to transport weights exceeding the recommended limits. Recently, wearable back-support exoskeletons have been designed to relieve the loading on the worker’s musculoskeletal (MSK) system from excessive lower back muscular forces and spinal joint loading (Hensel and Keil, 2019). Exoskeletons have been shown to improve the kinematics of the user, and reduce their low-back muscle activity or compressive forces during static bending or dynamic movement (Asbeck et al., 2014; De Looze et al., 2016; Toxiri et al., 2019; Kermavnar et al., 2021; Luger et al., 2021; Pesenti et al., 2021).

Back-support exoskeletons can be categorized into active and passive systems. Passive back-support exoskeletons rely on energy-storing mechanisms, such as spring or elastic components (Bosch et al., 2016; Lamers et al., 2018; Alemi et al., 2019). Despite being lightweight and non-bulky, passive back-support exoskeletons do not dynamically modulate the magnitude of the assistance to the specific lifting conditions, external loads, or the MSK characteristics of the user. Active exoskeletons provide assistive forces by means of actuators (series-elastic, serially connected linear actuators, hydraulic or pneumatic) (Hara and Sankai, 2010; Inose et al., 2017; Huysamen et al., 2018; Kim et al., 2024). Owing to their actuation and control loops, active back-support exoskeletons can provide a wider range of force profiles beyond spring-like behaviors. However, active back-support exoskeletons are typically heavy and have bulky and rigid frames and actuators, thereby hampering the wearer’s range of motion (Toxiri et al., 2018). Additionally, active exoskeletons require precise actuator colinearity with the joints of interest (*e.g.*, hip joint), thereby greatly increasing the donning time. These factors contribute to the general low acceptance of active exoskeletons in factory settings (De Looze et al., 2016).

Active soft exosuits are a specific category of active exoskeletons made of soft materials that interface with the human body, therefore offering a lightweight alternative to rigid exoskeletons (Asbeck et al.,2014). Exosuits typically transfer assistive forces to biological joints through actuated cable-driven mechanisms acting in parallel with the human musculature (Li et al., 2021; Chung et al., 2024). Thus, they integrate the flexibility and freedom of movement of soft devices with the versatile actuation mechanisms of active models. However, power transmission in soft exosuits is a challenge given the deformation experienced by biological tissues and physical interfacing materials (Yandell et al., 2017).

Current state-of-the-art controllers for back-support wearable robots aim to correct for gravitational effects on the trunk. An overview of exoskeletons and exosuits and their control strategies is provided in Supplementary Material A. These controllers determine the onset and magnitude of assistance based on the torso inclination, velocities, and accelerations. Therefore, they cannot adapt to changes in external loads lifted by users, for which they require additional sensors (Chen et al., 2018; Chung et al., 2024; Ding et al., 2024; Heo et al., 2020; Li et al., 2021; Luo and Yu, 2013; Quirk et al., 2023; Toxiri et al., 2018; Yang et al., 2019; Yao et al., 2019; Yong et al., 2019; T. Zhang and Huang, 2018). Trunk kinematics have been used to modulate the tension of cable-driven exosuit mechanisms, such that assistance was provided after a predefined threshold during the upward lifting motion (Luo and Yu, 2013; Li et al., 2021). Moreover, this approach cannot determine whether a load is being carried and requires additional modalities to switch assistance during the task or when in contact with loads. This includes the use of predefined inclination thresholds (Chen et al., 2018; Chung et al., 2024), electromyography (EMG), or pressure sensing (Hara and Sankai, 2010; T. Zhang and Huang, 2018), a combination of inclination and contact detection (Toxiri et al., 2018; Koopman et al., 2019), or using the kinematics of the lower limb (Yin et al., 2019) to detect contact with load or switch assistance levels. Finally, as these controllers do not include muscle activity, they cannot directly target the active component of lumbosacral compressive forces, which vary with interactions with the environment.

Thus, to the best of our knowledge, current control modalities for back-support exoskeletons and exosuits cannot continuously (1) target lumbosacral compressive forces directly during lifting, (2) adapt to user-specific MSK demands, or (3) adapt to the load being carried without *a priori* information. Nonetheless, in real-world occupational environments with unpredictable and unstructured movements, subject-specific and adaptive support to lifting dynamics or external loads can help improve the effectiveness of exoskeletons. In this context, an open challenge consists of determining the biomechanical effects of unknown external loading conditions on the MSK system of the user and to adapt respective support levels. Moreover, because of the nonlinear mapping between the torque and compressive loads, the influence of exosuits (informed by EMG-driven MSK models) on compressive loads is nontrivial and needs to be investigated.

EMG-driven MSK models offer a personalized approach to noninvasively estimate internal biomechanical metrics, such as muscle forces or joint loading. These models use personalized 3D anatomical representations of the MSK system, as well as physiological processes, such as muscle activation and contraction dynamics (Lloyd and Besier, 2003; Sartori et al., 2012a). Including experimentally measured EMG activity and human joint kinematics within EMG-driven models has been shown to estimate muscular forces dynamically, which can be translated into accurate internal forces at the ankle (Sartori et al., 2012a), knee (Gerus et al., 2013; Pizzolato et al., 2017), elbow (Manal et al., 2002), or lumbosacral (at the L5/S1) joints (Moya-Esteban et al., 2022). These models have been previously used within human–machine interfaces (HMIs) to control different exoskeletons and prostheses (Sartori et al., 2018; Lotti et al., 2020; Durandau et al., 2022). This includes providing ankle assistance during gait using a bilateral lower limb exoskeleton (Durandau et al., 2022), elbow assistance in a semi-soft upper-limb exosuit (Lotti et al., 2020), and in a unilateral wrist-hand prosthesis (Sartori et al., 2018). However, an EMG-driven approach, that directly estimates the personalized L5/S1 moments and subsequently, the compressive loads continuously, has not been used to control back-support exoskeletons or exosuits across different external loading conditions.

Therefore, in this study, we employed our previously validated large-scale (164 musculo-tendon units [MTUs]) real-time EMG-driven MSK modeling framework (Moya-Esteban et al., 2023) to estimate lumbosacral joint moments using wearable sensors. We extended the work by utilizing a neuro-mechanical model-based control (NMBC) strategy to provide support via a custom-made back-support cable-driven soft exosuit. The NMBC was designed to target the active component of the muscle activity surrounding the lumbosacral joint to determine the magnitude of the assistance provided by the soft exosuit during lifting tasks involving different weights. Although NMBC has no *a priori* knowledge of the lifted weight, we hypothesize that it could provide adaptive and subject-specific assistive forces, which are tuned to the specific lifting stage (box lifting/lowering) and loading conditions (lifted weight). We investigate the impact of our proposed model-based NMBC in reducing the muscle activity, lumbosacral joint moments, and cumulative compression forces along with an virtual impedance-based trunk inclination-based controller (TIBC). We hypothesize that compared to TIBC, our novel NMBC has the potential of providing versatile assistance levels in various lifting conditions under unknown external loads.

## 2. Methods

First, we describe the equipment (Section 2.1) required to employ our proposed EMG-driven MSK modeling pipeline, described in Sections 2.2 and 2.3. We then describe the structure of the two controllers analyzed in this article (Section 2.4) and the design of the back-support cable-driven soft exosuit (Section 2.5). Next, we describe the experimental procedures designed to evaluate our control strategies (Section 2.6) and the associated data analyses (Sections 2.7 and 2.8).

### 2.1. Subject instrumentation

Surface bipolar EMGs from rectus abdominis (RA; umbilicus level), iliocostalis (IL; 6 cm lateral to L2), longissimus thoracis pars lumborum (LTpL; 3 cm lateral to L1), and pars thoracis (LTpT; 4 cm lateral to T10) were acquired using Ottobock 13E400 electrodes (Ottobock SECo. KGaA, Duderstadt, Germany) at 1 kHz. Raw EMG recordings were filtered to obtain EMG linear envelopes: band-pass filter (30–300 Hz), full-wave rectified, and low-pass filtered (3 Hz). All filters were second-order Butterworth filters. Maximum EMG values from maximum voluntary contraction recordings (performed as described in (McGill, 1991)) were used to obtained normalized EMG linear envelopes.

Participants wore the MVN Link system (Movella, Enschede, The Netherlands), that is, an IMU suit. The MVN Link sensors were placed the following manufacturer’s guidelines on pelvis (middle point between posterior iliac spines), sternum, shoulders (scapula level), upper arms, and forearms. The “*Upper Body No Hands*” configuration was used to measure trunk inclination (defined as the orientation of the trunk at T8 with respect to the vertical axis) and L5/S1 joint flexion-extension angles at 40 Hz.

The experimental protocol was divided into experimental and calibration sessions (see Section 2.6). During the calibration session, we also recorded the 3D trajectories of 33 reflective markers (32 on participants and 1 on the upper edge of the lifted box) using a 12-camera Qualisys system (Qualisys Medical AB, Sweden) at 40 Hz. Marker placement was previously described in (Moya-Esteban et al., 2020). Marker data were used to compute joint angles and moments via inverse kinematics (IK) and inverse dynamics (ID), respectively, which were used to calibrate the subject-specific EMG-driven MSK models (see Section 2.3).

### 2.2. Multibody dynamics modeling

**Figure 1.**
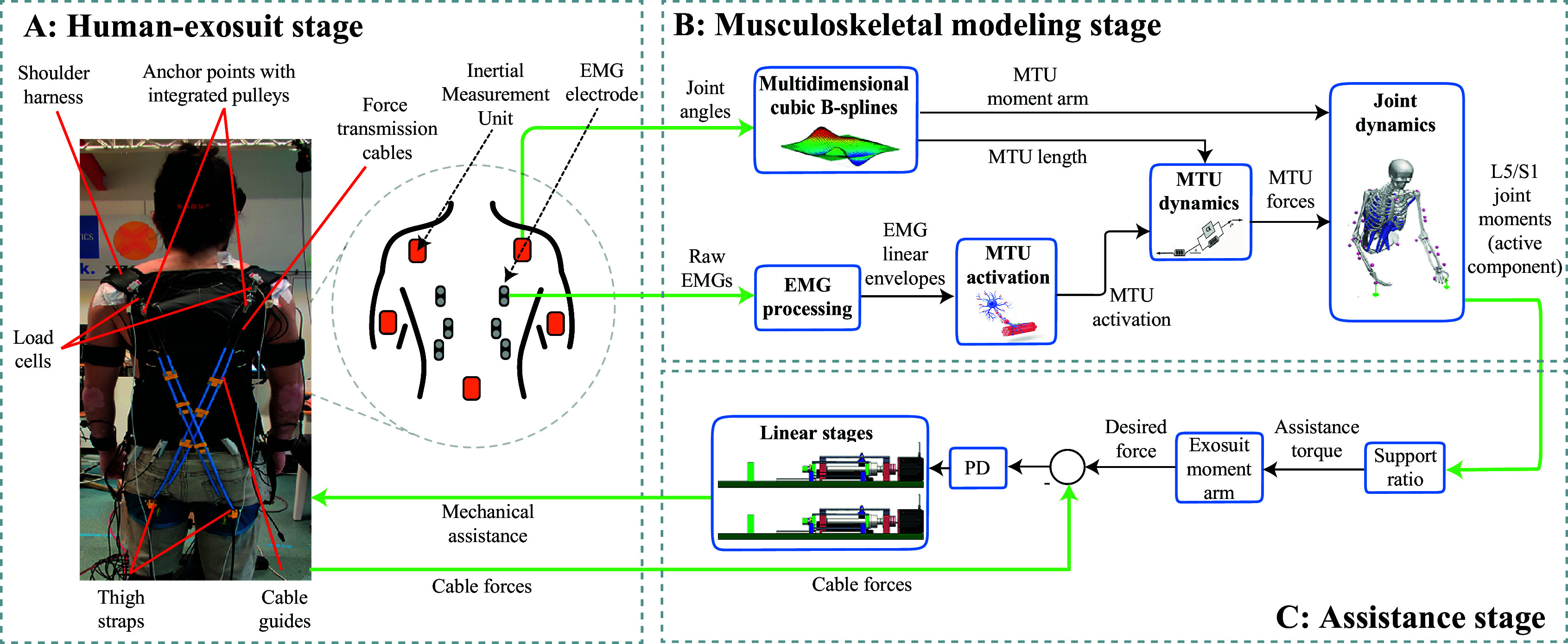
Extension of previous work (Moya-Esteban et al., 2023, Fig. 2) toward a NMBC to control the cable-driven soft exosuit. (A) In the human-exosuit stage, the main components of the exosuit are depicted. Sensors used to measure electromyographic signals (EMG) and joint angles (inertial measurement units [IMUs]) are represented. (B) In the MSK modeling stage, our previously validated EMG-driven MSK modeling framework (Pizzolato et al., 2015; Moya-Esteban et al., 2022, 2023) employed MTU lengths, moment arms and activations to obtain MTU forces, which were used to derive real-time L5/S1 flexion-extension joint moments. To do so, a multidimensional cubic B-spline block was used to estimate, in real-time, MTU lengths and three-dimensional moment arms, using joint angles as input. Raw EMGs were processed to obtain MTU-specific activations. The MTU dynamics block implemented a Hill-type MTU model which allowed estimating MTU forces. (C) The active component of real-time L5/S1 joint moments was scaled using a support ratio of 0.2 which was then divided by 0.08 m representing the moment arm of the force-transmission cables about the lumbosacral joint (Li et al., 2021).

A simplified version of the OpenSim lifting full-body model (Beaucage-Gauvreau et al., 2019) was used to compute ID L5/S1 joint moments, which were used for EMG-model calibration. Although this model has been explained in detail in our previous work (Moya-Esteban et al., 2023), here, we provide a brief summary. First, 3D marker data from a static recording were used to linearly scale the generic model to match the subject-specific anthropometry using OpenSim 4.1 (Delp et al., 2007). Based on the geometry of the scaled MSK models, we created a set of multidimensional B-spline functions (Sartori et al., 2012b). These functions allow for the real-time computation of MTU lengths and 3D moment arms as a function of L5/S1 flexion-extension joint angles (see Figure 1).

Prior to EMG-model calibration, 3D marker positions from dynamic box-lifting motions (see Section 2.6) were used to compute joint angles via IK. The force exerted by the box was applied on the participants’ hands if the vertical position of a marker on the box exceed a defined threshold. The magnitude of the forces was computed based on the weight being lifted. This information was only utilized for the calibration process. We neglected the inertial effects of the box and assumed that the weight was distributed equally across both hands. Subsequently, IK-derived joint angles and the estimated hand forces were utilized to compute the L5/S1 flexion-extension joint moments using a top-down ID approach.

### 2.3. Real-time EMG-driven MSK modeling

Relying on the MSK geometry of the scaled models, we used a subject-specific real-time EMG-driven MSK model of the trunk, using the calibrated EMG-informed neuromusculoskeletal toolbox for the real-time control of wearable robots (CEINMS-RT) (Pizzolato et al., 2015; Moya-Esteban et al., 2023; Sartori et al., 2024). EMG-driven models enabled the estimation of muscle-tendon forces using experimentally measured joint angles and EMGs, which can in turn be translated into joint kinetics via subject-specific geometrical models (see Section 2.2).

#### 2.3.1. Model calibration

For each participant, the EMG-driven model parameters that were calibrated included MTU maximum isometric force, tendon-slack length, and optimal fiber length for the 164 MTUs in the model. The base parameters were tuned between predefined boundaries using a simulated annealing algorithm (Goffe et al., 1994), which minimized the summed squared error between reference ID L5/S1 flexion-extension moments and EMG-based moments (computed using measured EMGs and B-spline-derived MTU lengths and moment arms). Therefore, a mapping between experimentally measured EMGs and MTUs in the adapted lifting full-body model was first established (see Supplementary Material B). The calibration was performed using one lifting repetition for each lifting condition (see Section 2.6).

#### 2.3.2. Model execution

Figure 1 depicts the calibrated EMG-driven models operated in an open loop using EMG and joint kinematics to estimate the L5/S1 joint moments. The MTU activation block allocated EMG linear envelopes to model MTUs (see Supplementary Material B) and processed the signals to account for the nonlinear EMG-force relationship (Buchanan et al., 2004). Personalized Hill-type MTU models were implemented in the MTU dynamics block, which included a representation of a stiff tendon, an active contractile element in parallel with a passive element, and a linear damper (Sartori et al., 2012a). Thus, passive force-velocity, passive, and active force-length relationships were used to model muscle fibers. Real-time computation of the MTU forces was performed in the MTU dynamics block using B-spline-derived MTU length activation, fiber contraction velocity, and pennation angle. Finally, MTU forces were projected onto the lumbosacral joint using the B-spline-derived MTU moment arms to obtain L5/S1 flexion-extension moments and compression forces.

### 2.4. Assistance stage

The exosuit linear stages (Section 2.5) communicated with the control computer via the EtherCAT real-time communication protocol. The real-time software (Section 2.3) and EtherCAT were executed on a Lenovo ThinkStation P620 (AMD Ryzen Threadripper PRO 3975WX, 3.50 GHz, 32 cores, 64 threads, 128 GB of RAM, and Windows 10). This computer executed the controller in TwinCAT 3 (Beckhoff Automation, Verl, Germany) in real time with a sampling frequency of 1 kHz. NMBC and TIBC were both implemented to determine the desired cable forces for both linear stages of our soft exosuit.

#### 2.4.1. Neuro-mechanical model-based controller

In this control mode, the active component of subject-specific lumbosacral joint moments derived from our EMG-driven modeling pipeline was sent to the exoskeleton low-level controller via Ethercat. Active moments were low-pass filtered (second-order Butterworth filter with cut-off frequency: 10 Hz), and multiplied by an assistance gain set to 20%. The gain was set after conducting a few tests with participants who provided feedback on whether the exosuit forces were assistive yet comfortable. Finally, the scaled active moments were time-delayed by 80 ms, which aimed at simulating the average electromechanical delay previously found for the lower back musculature. This delay was utilized in our previous work, where the average computation time of the EMG-driven pipeline was 27.2 ± 11.1 ms (Moya-Esteban et al., 2023).

#### 2.4.2. Trunk inclination-based controller

In this control mode, the assistance provided by the exosuit was proportional to the measured trunk inclination angle. The TIBC had no explicit knowledge of the external loading conditions (*i.e.*, weight of the lifted object). The control gain of TIBC was set to deliver assistive forces that were 20% of the forces resulting in lumbosacral joint moments over a moment arm of 0.08 m when the participant was holding a 5 kg weight at 30° torso angle. The moment arm captured the line of action of the exosuit cables with respect to the lumbosacral joint (Lamers et al., 2018). The TIBC was thus tuned to have similar assistance as NMBC when lifting the 5 kg weight.

### 2.5. Cable-driven exosuit design and control

The soft exosuit system provides assistive forces using force-transmission cables that are parallel to biological muscles (Li et al., 2021; Mohamed Refai et al., 2023). The exosuit was designed with the capability of providing assistance during both symmetric and asymmetric lifting tasks via the use of two force-transmission cables routed in a diagonal manner. Equal cable forces provided support during symmetric sagittal movements, and differential forces would be useful for asymmetric twisting movements. Here, we used the former modality.

To achieve this, two major elements were utilized: (1) the fabric-based interface worn by the user and (2) off-board actuation units that generated the assistive forces. The fabric interface of the exosuit was designed using neoprene fabric reinforced with 500D condura nylon material for increased tensile strength. The exosuit consisted of a shoulder harness and thigh straps with 3D-printed anchor points, which were sewn on to the fabric using high-strength polyester thread. To transmit the assistive forces to the user, steel cables with a diameter of 1 mm were utilized. One end of the cable was attached to the thigh harness, and the other end was guided through polyurethane cable guides up to the shoulder harness and passed through a pulley placed in series with a load cell (S610 45.35 kg, Strain Measurement Devices, CT, USA) integrated into the anchor points. The cable was then routed back to the thigh harness where it was connected to the off-board actuation unit using bowden sleeves.

The off-board actuation unit consisted of two ball-screw-driven linear stages (5 mm lead, MISUMI, Japan) which converted rotational motions generated using AC servo motors (AKM22C-BNCNC-00, Kollmorgen, VA, USA) (Figure 1c) to linear motions. The nut of the ball screw assembly was connected to a carriage, which was guided using a linear bearing for additional stability. The steel cables used to actuate the exosuit were connected to the carriage, which in turn transmitted the forces from the actuation unit to the user.

### 2.6. Experimental protocol

The experimental procedure was approved by the Natural Sciences and Engineering Sciences Ethics committee of the University of Twente (reference: 2020.38). A convenience sample of 10 participants (three females; age: 30 ± 2, height: 173 ± 7 cm, weight: 67 ± 9 kg) with no history of LBP were recruited for the study after having giving written informed consent.

The protocol was divided into calibration and experimental sessions. Participants wore the IMU suit and EMGs for both sessions, and additionally reflective markers for the calibration session. After subject instrumentation (Section 2.1), the maximum voluntary contraction trials were recorded. Then, participants lifted a box (w × d × h: 40 × 30 × 22 cm) using the stoop lifting technique (flexed trunk and extended but not locked knees) under two weight conditions (5 and 15 kg). Lifting repetitions consisted of (1) bending over to grab the box (which was resting on a 46.5 cm height table), (2) lifting the box until upright posture, (3) bending over to place the box, and (4) returning to upright posture. To control the movement speed, a metronome (30 beats-per-minute) indicated the start of each of the aforementioned phases.

In the calibration session, the participants performed one lifting repetition for each of the two weight conditions. In the experiment session, participants performed box-lifting repetitions in three conditions (without exosuit (NOEXO), with exosuit using NMBC or TIBC), and with two weights (5 and 15 kg). For each of the six experimental conditions, 10 lifting repetitions were recorded in sets of two, leaving a minute’s rest between sets. Lifting conditions were randomized, having both exosuit conditions (NMBC and TIBC) in one block, therefore avoiding recurrent exosuit donnings/doffings. Prior to data recording, the participants followed a 15-min familiarization period with both controllers.

### 2.7. Study analyses

We studied the modulation of the magnitude of the assistance forces for both NMBC and TIBC by analyzing human-exosuit work loops between the trunk inclination and normalized cable forces. Additionally, we evaluated the exosuit force tracking, that is, the difference between the desired and actual cable force (measured with the exosuit-embedded load cells) by computing root mean squared errors (RMSEs).

The mean and peak EMG activities for all measured muscles were summed to calculate the total reduction in EMG for both NMBC and TIBC with respect to the NOEXO condition. Similarly, the exosuit-induced reductions in (mean and peak) L5/S1 joint moments and compression forces were computed for the overall lifting cycle and during different phases of the lifting cycle, such as lifting, lowering, and erect stance. Furthermore, we computed the cumulative lumbosacral compression forces (kN  s) after 1, 5, and 10 dynamic lifting cycles. The cumulative forces were integrated via trapezoidal integration.

### 2.8. Statistical analyses

The normality of the residuals was confirmed using Shapiro–Wilk tests. Repeated-measures ANOVA was applied for assistive cable forces, EMG, L5/S1 moments, compression forces, and cumulative compression to find differences across three conditions (NOEXO, NMBC, and TIBC) and two weights (5 and 15 kg). Only results with significance are described. Statistical analyses were conducted using the SPSS software (IBM SPSS Statistics 26, SPSS Corporation, USA) as well as MATLAB R2024a.

## 3. Results

### 3.1. Human-exosuit work loops

**Figure 2.**
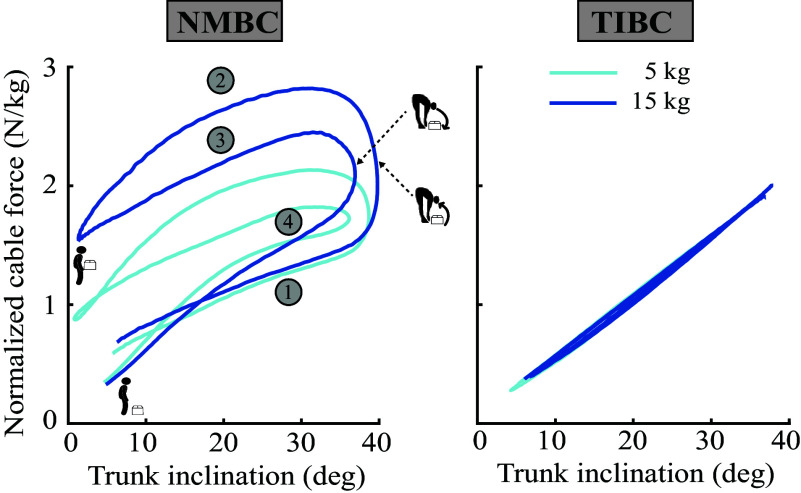
Human-exosuit work loops during assisted box-lifting tasks for neuro-mechanical model-based (NMBC) and trunk inclination-based (TIBC) controllers. Cable forces (summation of left and right cables and normalized to body weight) are plotted versus trunk inclination for 5 (light blue) and 15 kg (dark blue) conditions. The following lifting phases are indicated: (1) bending over to grab the box, (2) lifting the box, (3) bending over to place the box, and (4) going back to the erect standing posture. Additionally, box lift-off, box drop, and erect standing with and without box instants are depicted.

Figure 2 depicts the variation in assistive cable force while using either the NMBC or TIBC. The NMBC adapted the exosuit assistive forces during the lifting cycle compared to TIBC (see Supplementary Material C for subject-specific work loops). The differences in cable forces between 5 and 15 kg for NMBC was significant (p < 0.01). We see that NMBC was able to adapt the assistance as soon as the user lifted the box. During lifting at 20° trunk inclination, the average NMBC-derived assistance force was 1.96 ± 0.52 N/kg and 2.64 ± 0.72, for 5 and 15 kg, respectively. Further, we see that during 20^2^ trunk inclination, NMBC provided higher assistance during the upward box-lifting motion than for the downward box-lowering motion (5 kg: 1.58 ± 0.50 N/kg; 15 kg: 2.20 ± 0.62 N/kg). The cable forces between 5 and 15 kg were not significantly different for TIBC (5 kg: 1.08 ± 0.46 N/kg; 15 kg: 1.08 ± 0.49 N/kg).

Overall, NMBC’s adaptation to the movement direction resulted in a positive work loop. The area under the curve in Figure 2 was 0.27 N rad/kg and 0.32 N rad/kg for NMBC for the 5 and 15 kg cases, respectively. However, TIBC only performed a network of 0.06 N rad/kg and 0.05 N rad/kg for the 5 and 15 kg cases, respectively.

### 3.2. Exosuit force tracking

**Figure 3.**
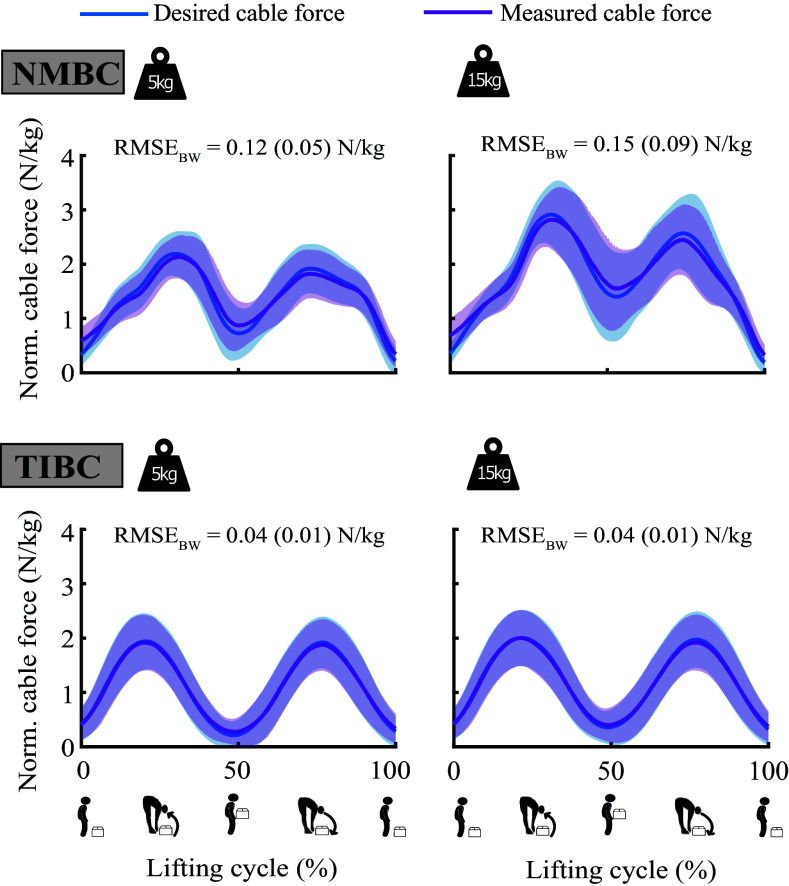
Exosuit force tracking for neuro-mechanical model-based controller (NMBC) and trunk inclination-based controller (TIBC) for 5 and 15 kg (left and right columns, respectively) lifting conditions. Measured and desired cable forces depict the summation of left and right cables. Solid lines indicate mean values across participants, and shaded areas correspond to ± 1 standard deviation. Root mean squared errors normalized to body weight, 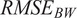 (N/kg), are shown for each condition. Values within parenthesis indicate the standard deviation.

Figure 3 shows the small differences between the desired and measured cable forces, as indicated by the RMSE. Similar measured cable forces were found around 25% of the box lifting for TIBC during 5 and 15 kg conditions (1.92 ± 0.51 N/kg and 2.00 ± 0.52 N/kg, respectively). However, NMBC exhibited a modulation of the measured forces, where significantly higher forces were measured during the high-weight condition (5 kg: 2.13 ± 0.40 N/kg; 15 kg: 2.82 ± 0.53 N/kg). Similarly, the cable forces while holding the weight at the erect stance (50% of the lifting cycle) were modulated for NMBC (5 kg: 0.87 ± 0.45 N/kg; 15 kg: 1.55 ± 0.75 N/kg), but not for TIBC (5 kg: 0.27 ± 0.24 N/kg; 15 kg: 0.40 ± 0.31 N/kg). Furthermore, support provided by TIBC during the erect stance while holding weight was similar to the assistance provided at the beginning and end of the lifting cycle, where the participant was not holding the weight (Figure 3).

### 3.3. Exosuit-induced biomechanical reductions

**Figure 4.**
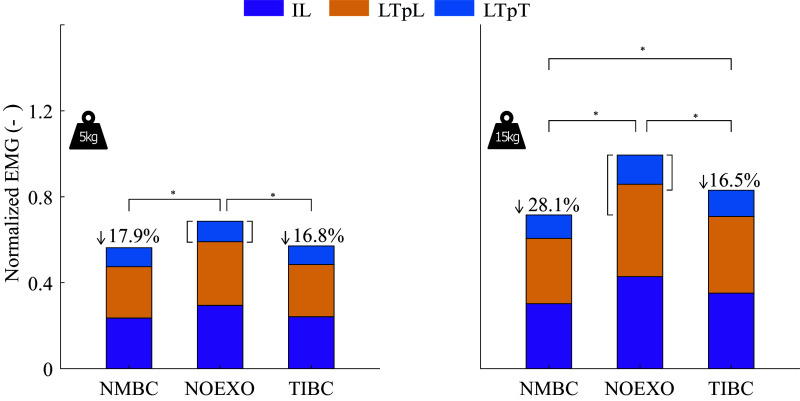
Normalized EMG values (averaged across the complete lifting cycle and participants) for iliocostalis (IL), longissimus thoracis pars lumborum (LTpL), and pars thoracis (LTpT), for NOEXO, neuro-mechanical model-based control (NMBC), and trunk inclination-based control (TIBC) conditions. The bars consist of blocks that depict the summation of left and right muscles. Numerical values with downward facing arrow indicate overall percentage of EMG reduction with respect to the NOEXO condition. Statistically significant differences are indicated by horizontal brackets with * ( 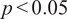 ).

The mean and peak muscle activities were significantly different between the 5 and 15 kg lifting conditions. Figure 4 shows the differences in mean EMG activity during the whole lifting cycle. The mean EMG activity during 5 and 15 kg lifting was significantly reduced for both NMBC and TIBC, with respect to NOEXO conditions). Both controllers reduced muscle activity similarly for 5 kg lifting conditions (NMBC: 0.12 ± 0.12; TIBC: 0.12 ± 0.08). However, when lifting 15 kg, NMBC showed larger mean EMG reductions (0.28 ± 0.17), compared to TIBC (0.16 ± 0.15). The differences in peak EMG activity were also studied and are shown in Supplementary Material D. Only TIBC significantly reduced peak muscle activity compared to NOEXO condition for both weights lifted. Moreover, TIBC showed larger reductions (14.6%) than NMBC (9.7%) when lifting 5 kg. NMBC only showed significant reductions in peak EMG activity compared to NOEXO for the 15 kg condition.

Supplementary Material E demonstrates the differences in reductions of peak EMG activity when considering specific phases of the lifting cycle. During the erect stance (40–60% of the lifting cycle), significant reductions in peak EMG activity were found between NOEXO and both controllers, irrespective of the weights lifted. Similarly, for both weight conditions, NMBC reduced the peak activity more than TIBC. However, during the lifting phase of the cycle (25–40% and 75–100%), only TIBC reduced the peak EMG activity for both weights. During the lowering phases (1–25% and 60–75% of the cycle), both NMBC and TIBC showed reductions in muscle activity compared to the NOEXO condition. This was true for both weight conditions. Overall, the largest reductions in peak EMG when using NMBC were observed during the erect phase (28.5%) and when using TIBC was observed during lifting phase (18.3%).

**Figure 5.**
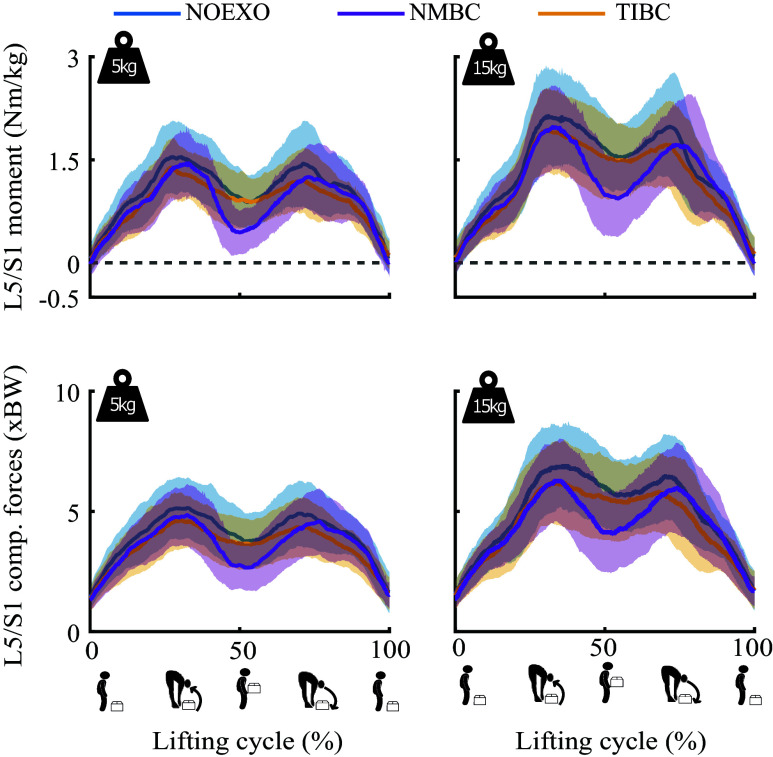
L5/S1 flexion-extension joint moments (normalized to participant body weight) and L5/S1 compression forces (expressed as times body weight) for 5 and 15 kg weight conditions. Time profiles are shown for the conditions without exosuit (NOEXO), neuro-mechanical model-based control (NMBC), and trunk inclination-based control (TIBC). Solid lines indicate mean values across participants, and shaded areas correspond to ±1 standard deviation.

**Figure 6.**
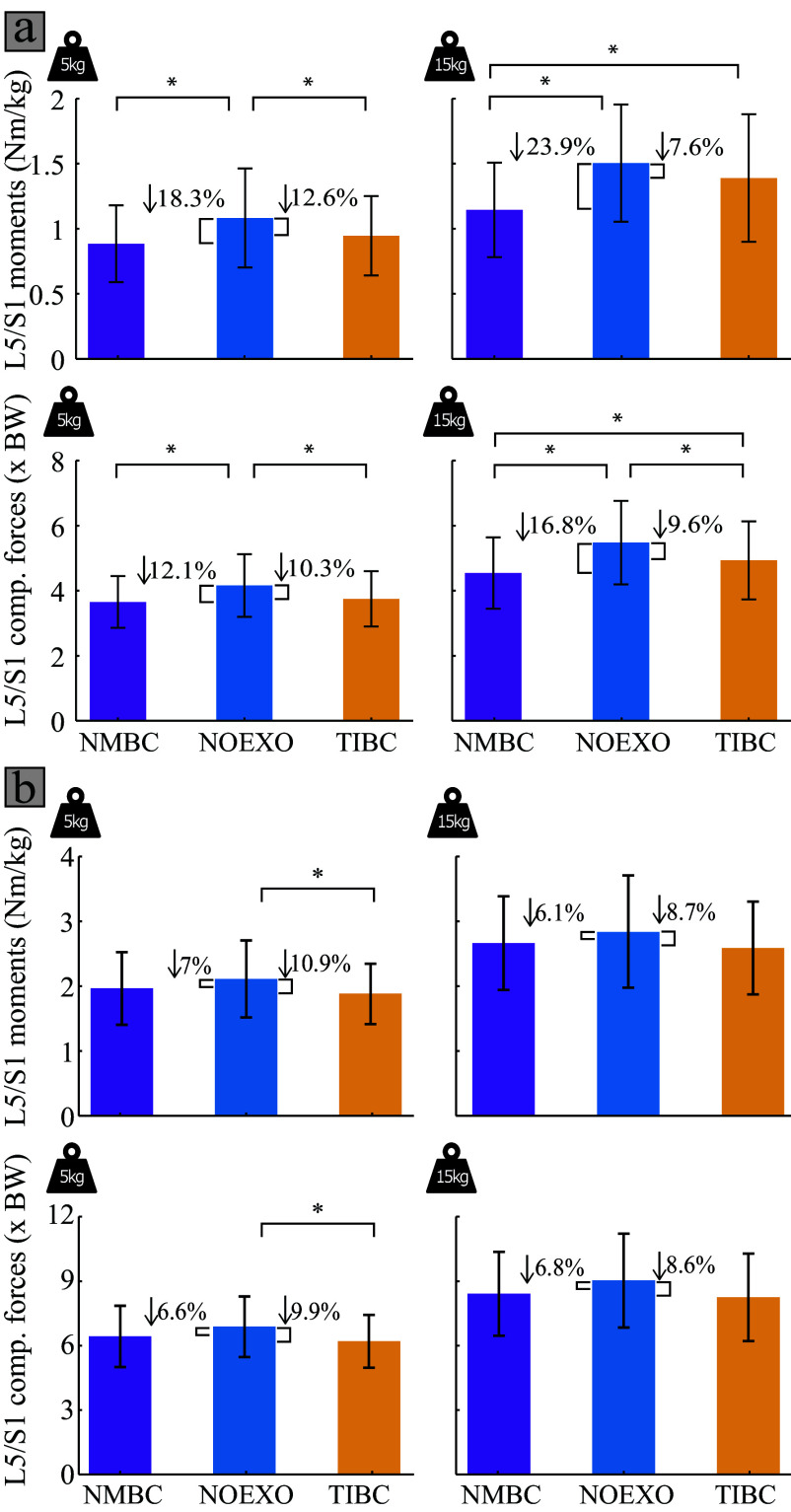
L5/S1 flexion joint moments and compression forces for 5 and 15 kg weight conditions, and NOEXO, neuro-mechanical model-based control (NMBC), and trunk inclination-based control (TIBC) conditions. (a) Mean and (b) peak moment and compression force values are computed across participants and the complete lifting cycle. Numerical values with downward facing arrow indicate the overall moment or compression force reduction with respect to NOEXO condition. Statistically significant differences are indicated by horizontal brackets with * ( 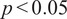 ).

Figure 5 depicts the average lumbosacral joint moments and compression forces for all experimental conditions. Figure 6 shows the reductions in moment and compression force reductions by each of the controllers. Figure 6a summarizes the differences in mean values, whereas Figure 6b shows the differences in peaks for both moments and compressive forces. When considering the mean moments and compressive forces during the 5 kg lifting, both NMBC and TIBC show reductions compared to the NOEXO condition. While lifting 15 kg, NMBC showed reductions compared to NOEXO as well as to TIBC for both mean moments (NOEXO: 1.50 ± 0.45 Nm/kg, NMBC: 1.14 ± 0.36 Nm/kg, and TIBC: 1.39 ± 0.49 Nm/kg) and for compressive forces (NOEXO: 5.46 ± 1.29 BW, NMBC: 4.54 ± 1.09 BW, and TIBC: 4.94 ± 1.19 BW). TIBC showed reductions only for mean compressive forces during the 15 kg lifting condition. However, when considering the peak moments and compressive forces, there were significant differences only when supported by TIBC (moments: 1.71 ± 0.42 Nm/kg, and compressive forces: 5.64 ± 1.12 BW) compared to the NOEXO (moments: 1.92 ± 0.54 Nm/kg, and compressive forces: 6.25 ± 1.28 BW) during the 5 kg lifting condition. Overall, the reductions by NMBC were larger for mean rather than peak moments and compressive forces, whereas the magnitude of reductions by TIBC were similar across the mean and peak metrics.

In Supplementary Materials F and G, we show the reductions, respectively, for peak and mean moments and compressive forces during specific phases of the lifting cycle. During the erect stance, NMBC reduced both mean moments and compressive forces compared to the NOEXO as well as TIBC. This was true for both weight conditions. However, while comparing peak metrics, NMBC only showed reductions with respect to NOEXO during 15 kg lifting. TIBC showed reductions in peak compressive forces when considering the 15 kg lifting conditions. During the lifting phase, mean compressive forces were reduced by both NMBC and TIBC compared to NOEXO conditions when lifting 5 kg, whereas TIBC showed reductions in peak compressive forces, irrespective of the weight carried. During the lowering phase, NMBC showed reductions in mean moments and compressive forces for the 5 kg condition compared to the NOEXO condition. TIBC showed reductions in peak and mean moments only when lifting the 5 kg weight.

**Figure 7.**
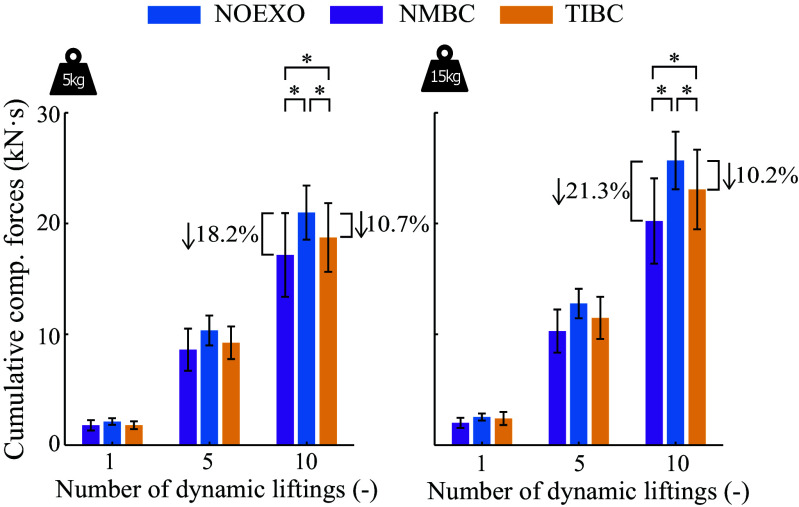
Mean cumulative L5/S1 joint compression forces after 1, 5, and 10 symmetric stoop box-liftings, for 5 and 15 kg weight conditions, and NOEXO, neuro-mechanical model-based control (NMBC), and trunk inclination-based control (TIBC) conditions. Numerical values with downward facing arrow indicate the overall cumulative compression force reduction with respect to NOEXO condition. Statistically significant differences are indicated by horizontal brackets with * ( 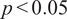 ).

The average cumulative lumbosacral compression forces were reduced after several dynamic box-lifting repetitions, by both NMBC and TIBC with respect to the NOEXO conditions (see Figure 7). After ten 15 kg stoop lifting cycles, the NOEXO condition resulted in total compressive loads of 25.7 ± 2.60 kN  s. This was reduced to 20.23 ± 3.85 kN  s and 23.07 ± 3.59 kN  s for NMBC and TIBC, respectively. For both 5 and 15 kg conditions, the cumulative compression force reduction for NMBC was significantly greater than that achieved by TIBC.

## 4. Discussion

We presented for the first time a subject-specific NMBC strategy for a cable-driven soft exosuit assisting dynamic box lifting tasks. NMBC provided exosuit assistance forces as a direct function of the active component of biological lumbosacral joint moments. NMBC showed most reductions when assessing the mean of the biomechanical metrics studied, or when assessing the lifting phase when the participant held the box in an erect stance. Further, we show the modulation of lumbosacral compressive forces as a function of adaptive assistance provided by an active soft exosuit. We see a reduction in cumulative compression forces by 21% during the tasks tested. The ability to personalize assistance to each user could potentially provide better interfacing of exosuits with the user.

Subject-specific adaptations of our NMBC were achieved through an optimization-based calibration performed for each participant. In our previous work, we had demonstrated the ability of our models to provide internal spinal forces with a computation time below the muscle electromechanical delay despite the large amount (164) of MTUs used (Moya-Esteban et al., 2023). This is a critical factor for enabling adaptive and intuitive control of assistive devices. A perceivable desynchronization (*e.g.*, resulting from computation times above the electromechanical delay) would lead to unstable assistance forces which may potentially counteract human movements and create instability. Our large-scale EMG-driven models allowed for estimating lumbosacral moments in a computationally efficient manner, allowing for intuitive control.

In this study, we also assessed the performance of a trunk inclination-based approach (TIBC), in which assistive forces were directly proportional to participants’ trunk inclination angle (Chung et al., 2024; Li et al., 2021; Yao et al., 2019; T. Zhang and Huang, 2018). The reductions in biomechanical metrics for both TIBC and NMBC were observed. Overall, NMBC reduced the mean and peak EMG when considering either the complete lifting cycle (15 kg) or the erect stance (both 5 and 15 kg). TIBC showed reductions across the lifting and lowering phases of the lifting cycle but not during the erect stance (Supplementary Material E and F). Additionally, over the whole lifting cycle, NMBC did not influence the peak moments and compressive loads compared to the NOEXO or TIBC (Figure 6b). However, TIBC showed reductions for these metrics with respect to the NOEXO condition for the 5 kg condition. When considering only the lifting phase of the tasks, TIBC significantly reduced the peak compressive forces with respect to the NOEXO condition for both weights. During the lowering phase, TIBC also reduced peak moments with the 5 kg.

Significant EMG reductions (17%) with respect to NOEXO conditions were achieved by both TIBC and NMBC for 5-kg dynamic liftings (Figure 4). However, for heavy weight conditions, the average EMG reduction provided by NMBC (28%) was significantly greater than those provided by TIBC (16%). This suggests the adaptability of NMBC when providing assistive forces to the specific lifting conditions. These reductions are in line with literature (De Looze et al., 2016; Kermavnar et al., 2021). In (Li et al., 2021), EMG reductions for the lumbar erector spinae muscle decreased as the weight of symmetric lifting tasks increased (45.2, 37.4, and 30.8% for 6.8, 15.9, and 22.7 kg, respectively). This behavior was likely a consequence of the constant assistive forces across the explored weight conditions. Similar EMG reductions, as those found for NMBC, were observed for previous active (Heo et al., 2020; Chung et al., 2024) and passive (Abdoli-E and Stevenson, 2008) back-support exoskeletons. Nonetheless, some rigid back-support exoskeletons like the Active Pelvis Orthosis, achieved larger EMG reductions with similar weights (Chen et al., 2018). Median EMG activity of the lumbar and thoracic erector spinae was reduced by 30% and 34.1%, respectively, when lifting a 5 kg box. A comparison of our exosuit device with previous exoskeletons is challenging due to differences in lifting conditions (technique, lifted weight, exoskeleton weight) and the type of assistance provided by the exoskeleton, that is, typically rotational moments around the hip joints. For a total of 10 participants, we demonstrated reductions in the mean muscle activity of the back extensor musculature, as well as the associated lumbosacral joint moments and compression forces by NMBC. The subject-specificity of NMBC has not been implemented in other current controllers, where biomechanical parameters are typically tuned to be similar across participants (Yang et al., 2019).

Providing assistive forces during the erect standing might be beneficial in minimizing the creep loading that leads to risk of LBP within occupational environments (Gallagher and Huangfu, 2019). When participants reached the upright standing pose while holding the box (50% lifting cycle), TIBC-derived cable forces decreased to initial values (erect standing with no weight) as a result of the near-zero trunk inclination angle. On the contrary, NMBC provided significant assistive forces in the proximity of erect standing while holding weight (*i.e.*, end of box-lifting and beginning of box-lowering), as a consequence of the increased biological lumbosacral joint moments generated by the weight of the box. This effect was especially evident during 15 kg conditions (Figures 2 and 3). As seen in Figure 5, the lumbosacral compression forces at upright stance with 15 kg weight remained as high as 5.5 times the participant’s body weight (or 3700 N), for NOEXO conditions. This mechanical load is above the recommended limit specified by industry safety and ergonomics guidelines set by the Revised NIOSH lifting equation (Waters et al., 1993). Compared to TIBC, NMBC managed to reduce erect standing loads up to 25.5% to values around 2700 N (Figures 5 and 6b). TIBC rather showed consistent reductions across mean and peak biomechanical metrics. NMBC must be further tuned to address the maximum muscle activity, and resulting compressive forces acting on the lumbosacral joint.

Although NMBC offers higher cable forces (Figure 3) performing a positive work (Figure 2) during the lifting cycle, the timing of the assistance was not tuned to target peak moment reduction, especially when the user reaches maximum flexion. The cable forces from NMBC reach their peak later than TIBC. A possible explanation is the 80 ms delay that was hard-coded into NMBC controller, which could be optimized in later studies. Moreover, as NMBC utilizes an EMG-driven model, we have access to both the timing as well as amplitude of the biological moments during the movement ahead of time. This is due to the estimation of biological torques from muscle activity, which manifested before (50–100 ms) the resulting lumbar joint biomechanical moment. Here, we chose to tune the electromechanical delay to synchronize the exosuit-generated moments with human biological moments estimates, which has been a successful strategy in reducing muscle effort during walking in a variety of conditions (Durandau et al., 2022). However, from other studies, we know that it might be beneficial to provide exoskeleton torques earlier or later than biological torques. Reductions of metabolic cost of walking while wearing an exosuit have been achieved by providing delayed biological hip and knee torque estimates (Molinaro et al., 2024). Providing corrective responses using an ankle exoskeleton faster than biological responses improved standing balance performance (Beck et al., 2023; Eveld et al., 2024). Thus, the NMBC estimates used in this study could be delayed less than the currently modeled EMD to reduce the peak biomechanical metrics. Introducing a human-in-the-loop approach (J. Zhang et al., 2017) could help in identifying these optimal timing strategies for lifting. These aspects must be studied in future work.

TIBC adapted to the user’s kinematics, but did not generalize or modulate to the lifting or lowering phases (Yang et al., 2019; Quirk et al., 2023; Chung et al., 2024). Tuning TIBC to a higher assistance could potentially have higher benefits; however, this requires adapting the controller to the magnitude of the weight being carried (Koopman et al., 2019; Chung et al., 2024), as well as the phases of the lifting cycle. In the case of NMBC, moment estimations were solely based on trunk EMG and lumbosacral joint kinematics, therefore not requiring any previous assumption on the lifting conditions. NMBC was not only adapted to the user’s MSK demands during dynamic tasks, but also the weight being lifted.

Comparatively, alternative techniques that modulate assistance provided based on a pre-defined inclination angle (Chen et al., 2018; Chung et al., 2024) requires *a priori* knowledge of the threshold for each subject and lifting tasks. Moreover, the use of EMG/pressure sensors to switch assistance profiles or detect when the user comes in contact with a load (Hara and Sankai, 2010; Koopman et al., 2019; T Zhang and Huang, 2018) can be beneficial, but might fall short in more dynamic movements of the lifting. Additionally, NMBC shows advantages in the neighborhood of erect standing while holding weight, which is not directly targeted by state-of-the-art back-support exoskeletons. This reduction in the overall cumulative lumbosacral compression force could contribute to minimizing the risks of low-back pain in occupational environments (Gallagher and Huangfu, 2019).

Our exosuit-generated assistive forces between the thighs and the shoulders via two cable-driven actuators with an assistance tracking RMSE below 15% of the target cable force (Figure 3). The low RMSE suggests that the loss in the transmission of desired forces between the exosuit and user was sufficient to provide assistance during the task. Nonetheless, detailed interfacing models could help us improve the efficiency of the controller (Yandell et al., 2017; Scherb et al., 2023). The cable forces were modulated by the proposed NMBC using trunk EMGs and lumbosacral joint kinematics as input. Without *a priori* information of the lifted weight or the lifting technique, assistive cable forces were adapted to the mechanical demands of the dynamic box-lifting task (Figures 2 and 3) and participants (see Supplementary Material C). This control paradigm is well-suited for occupational environments (e.g., factories or warehouses) with predominant manual material handling tasks. Here, the mechanical loading of the MSK system constantly varies according to the workers’ lifting preferences and MSK conditions (such as the presence of low-back pain, MSK strength, or muscle fatigue); the working task; the characteristics of the lifted object, or ergonomic factors.

The NMBC cable forces were determined by linearly scaling the active component of the biological lumbosacral moments. By doing so, we aimed at relieving the MSK system from actively generated lumbar loading. However, even in box-lowering phases, wherein passive force generation mechanisms play a central role (Moya-Esteban et al., 2022), the magnitude of active moments was significant, generating therefore considerable cable forces (see Figure 3). Nevertheless, we did not find an increase of EMG activity in the antagonistic muscle group (RA). This suggests that users did not attempt to counteract the exosuit forces by co-activating the trunk musculature. Instead, participants likely leaned their body weight on the device during the box-lowering stages, resulting in predominant EMG reductions for the lumbar musculature (Figure 4).

So far, from current studies, it was unknown how lumbosacral compression forces are modulated given adaptive support from a soft exosuit. For the first time, we demonstrated that after 10 dynamic liftings of a 15 kg weight, our proposed NMBC significantly reduced cumulative lumbosacral joint loading by 21.3% with respect to unassisted conditions. NMBC showed larger reductions compared to the kinematic-based TIBC, where cumulative reductions were 10.2% across participants. Cumulative compression forces have been shown to contribute to low-back pain development (Norman et al., 1998; Gallagher and Huangfu, 2019). However, we observe that NMBC does not reduce the peak biomechanical metrics as much as TIBC. Thus, these results suggest that a neuro-mechanical model-based paradigm has the potential to reduce the impact of low-back pain (and related MSK disorders) in occupational scenarios where repetitive manual material handling is a major component of workers’ daily tasks. Further fine-tuning of the controller can also help reduce the influence of peak loading on the user.

### Limitations and future research

Healthy participants were included in this first study. Nonetheless, we hypothesize that NMBC controllers would also be beneficial for participants with LBP. The EMG-driven MSK model would reflect the changes in muscle activity for these participants and can be used to provide the required assistance.

NMBC was tested only during stoop lifting, which is not commonly recommended for material handling. Squatting while wearing the exosuit in its current state caused excessive cable friction limiting the range of motion of the users. This was due to the use of a tethered setup. The wearability of the setup could be improved by using an on-board actuator (Li et al., 2021; Chung et al., 2024). Although we did not evaluate our NMBC under realistic (semi)-squat lifting conditions, our EMG-driven MSK methodology has been previously validated for squatting tasks (Moya-Esteban et al., 2023), which suggests that similar adaptive assistance profiles may be achieved under real-life factory-inspired lifting techniques. Additionally, studies need to be performed to assess the impact of the controller during more real-life tasks, such as walking or turning, which are closer to an industrial setting.

The current setup cannot be used as is in a practical setting. The IMUs and EMGs used to inform NMBC must be rendered wearable for actual practice. Nine IMUs were used to estimate the L5/S1 joint. However, this could be reduced to just two sensors around the lumbosacral region (Brouwer et al., 2024). Alternatively, markerless approaches to estimate the pose of the user could be explored (Wang et al., 2021). Eight EMG sensors were used to measure the muscle activity. The EMG sensor placement required sufficient preparation of the skin surface of the participant and sufficient taping to ensure that the sensors remained in place. This is not practical. Techniques that can extract muscle activity from a large grid of textile electrodes can improve the wearability of using EMG sensors (Simonetti et al., 2022). Alternatively, as the muscle activity of the trunk is highly synergistic, the number of EMG sensors required can be reduced using inverse synergy-based approaches (Rook et al., 2024). Studies that explore these issues can improve the wearability of the setup presented in this study.

Despite the advantageous biomechanical effects provided by our exosuit (at EMG, joint moment and compression force levels), the presented reductions were likely underestimated due to a mismatch in timing of NMBC and the lack of familiarization of the participants to the exosuit assistance (Diamond-Ouellette et al., 2022). After exoskeleton donning, participants only followed a 15-min practice period to become familiar with the assistance provided by NMBC and TIBC. Future research will aim at assessing the effect of dedicated exosuit training sessions on the biomechanical impact of our device. Additionally, in this study, a support ratio of 20% was used to determine the cable forces from the active component of the lumbosacral moments. The current exosuit design did not allow us to explore higher support ratios, and this should be explored in future designs. Nonetheless, this should be considered carefully alongside the comfort of the exosuit, which is a key element for adoption in industrial settings.

The validity of our EMG-driven modeling framework to estimate lumbosacral joint moments and compression forces has been previously evaluated in a study with similar participants and experimental conditions (Moya-Esteban et al., 2023). Hence, quantifying the estimation accuracy of our models did not constitute an objective of this study. In order to improve the comfort of participants, we excluded motion capture recordings from the experimental session, which precluded the estimation of the 3D forces and moments exerted by the lifted box on the subjects. These forces are required for accurate ID and joint reaction analyses. Hence, the computed L5/S1 compression forces were likely underestimated. Nonetheless, the maximum contribution of a 15 kg weight box to compression forces is below 150 N, which represents only 4.5% of the average compression force during the NOEXO conditions. Furthermore, the underestimation error did not vary between conditions, which supports the assumption that not including box-derived forces and moments may not have had a significant effect on the biomechanical comparison between the exosuit conditions. Moreover, the moment arm of the force-transmission cables was set to be a constant (8 cm) throughout the dynamic task. This was lower than the values estimated using imaging studies (Lamers et al., 2018; Quirk et al., 2023; Rodzak et al., 2023). Furthermore, changes in the pose of the user during a task can vary how the exosuit wraps around the back. Using a dynamically varying moment arm can improve the estimates of lumbar moment, which is used to provide assistance to the user.

Finally, the present study was conducted under controlled conditions in a laboratory setting. Previous research has highlighted the need of testing exoskeletons in real-life settings, given the presence of realistic lifting techniques and the importance of assessing user acceptance (Kermavnar et al., 2021). Future research will explore mechanical adaptations to the soft exosuit design in order to enable realistic lifting motions of occupational settings. These factors will contribute to the translation of this robotic technology to real-life workplaces.

## 5. Conclusion

We presented an NMBC for back-support exosuits that uses real-time EMG-driven MSK models to derive subject-specific lumbosacral joint moments and compressive forces. NMBC directly targeted the active components of muscle forces surrounding the lumbosacral joint, which contribute to the moments and compressive forces. As a result, we demonstrate (1) a soft exosuit that continuously adapts to tasks as well as external loads without *a priori* knowledge of the weights, making it task- and load-agnostic, and (2) the influence of the exosuit on the lumbosacral compressive forces. We show that the cumulative lumbosacral compression forces can be significantly reduced (up to 21%) as a result of our model-based approach. NMBC showed most reductions in mean metrics during the erect stance while holding weight, and the peak values in limited cases. In contrast, a simple inclination-based controller was more suitable to reduce the impact of peak metrics during lifting. Due to its use of an EMG-driven model, NMBC can be further optimized to reduce the peak and mean metrics or muscle fatigue. This study constitutes a first step for the development of robust and versatile HMIs for robotic exoskeleton control. Further studies are required to fine-tune this novel controller and improve its wearability in practice.

## Supporting information

Moya-Esteban et al. supplementary material Moya-Esteban et al. supplementary material

## Data Availability

Data can be made available to interested researchers upon reasonable request by email to the corresponding author.

## Supplementary material

The supplementary material for this article can be found at http://doi.org/10.1017/wtc.2025.3.
