# Supplementary material for: Soft back exosuit controlled by neuro-mechanical modeling provides adaptive assistance while lifting unknown loads and reduces lumbosacral compression forces
Source: Wearable Technol. 2025 Feb 24;6:e9. doi: 10.1017/wtc.2025.3 (PMC11894419; doi:10.1017/wtc.2025.3)
Supplement: Moya-Esteban et al. supplementary material [file S2631717625000039sup001.pdf]

Supplementary Materials

A: An overview of back support exoskeletons and exosuits

Table A1: An non-exhaustive overview of the controllers used for active back support devices

Exoskeleton/Exosuit	High-level Control Strategy used	References
AWN-12 Model Y	Motion-based: sensors on the waist detect movement. Support during lifting, gravity compensation during lowering and no support during walking.	(Toxiri et al., 2019)
CRAY X – 4th gen	Speed-dependent control. 2 working modes (static, agile) and possibility for basic parameters' setting.	(Govaerts et al., 2024; Schwartz et al., 2022; Walter et al., 2023)
Robo-Mate MK2-b	Torque proportional to trunk angle and load detection based on EMG bracelet around the forearm.	(Koopman et al., 2019; Toxiri, Calanca, et al., 2018; Toxiri, Koopman, et al., 2018)
APO	Control based on adaptive dynamic movement primitives.	(Chen et al., 2019; Giovacchini et al., 2015; Lanotte et al., 2021)
HAL lumbar support	Two subsystems working in parallel: cybernetic voluntary control (CVC), EMGs for intention detection, cybernetic autonomous control (CAC), gravity compensation.	(Hiromasa Hara & Yoshiyuki Sankai, 2010; Miura et al., 2018)
LAD	Finite state machine on trunk inclination and pelvis joint angle. 3 states: standing, lowering, and lifting.	(Lee & Kim, 2019)
ExoBack v1	Motion-based. Different pre-defined modalities available and possibility for basic parameters' setting.	(Schwartz et al., 2022)
H-Wex v2	Inclination-based. Virtual spring mode and gravity compensation, with possibility for basic parameters' setting	(Hyun et al., 2020; Ko et al., 2018)
WPAD	A displacement sensor is triggered by the leg movement to	(Yin et al., 2019)

	open solenoid valves and contract the pneumatic muscles.	
Custom exoskeleton 1	Motion-base: finite state machine on encoders torque and angles. 3 states: lifting, lowering and no load. Sensorized gloves to detect external objects.	(Zhang & Huang, 2018)
AB-Wear III	Artificial muscles are directly activated by the user via a button.	(Yamanaka et al., 2021)
Hip Active Exoskeleton	Motion-based. It includes a finite state machine with 6 states: semi-squat, upright, right front and back swing, left front and back swing.	(Wei et al., 2020)
Portable Pneumatic Exoskeleton	Motion-based on measured hip angles and information from load cells in series with the cylinders' rods.	(Heo et al., 2020)

B: Electromyograms-musculotendon unit mapping

TABLE B1: MEASURED ELECTROMYOGRAMS (EMG) AND MODEL MUSCLE-TENDON UNIT MAPPING

**Muscle group in adapted lifting-full body model**	**Measured EMG**
Rectus abdominis, external and internal obliques	Rectus abdominis
Iliocostalis pars lumborum	Iliocostalis
Longissimus thoracis pars lumborum	Longissimus thoracis pars lumborum and multifidus
Longissimus thoracis pars thoracis and iliocostalis pars thoracis	Longissimus thoracis pars thoracis

Table B1 shows the muscle-tendon unit groups in the adapted lifting full-body model and the associated experimentally measured bipolar EMGs. Muscle-tendon units belonging to latissimus dorsi, quadratus lumborum and psoas major muscle groups were not driven by EMGs, therefore, solely contributing with the passive musculotendon force component.

C: Subject-specific human-exosuit work loops

Fig. C1 depicts human-exosuit work loops for each participant, NMBC, TIBC and weight conditions.

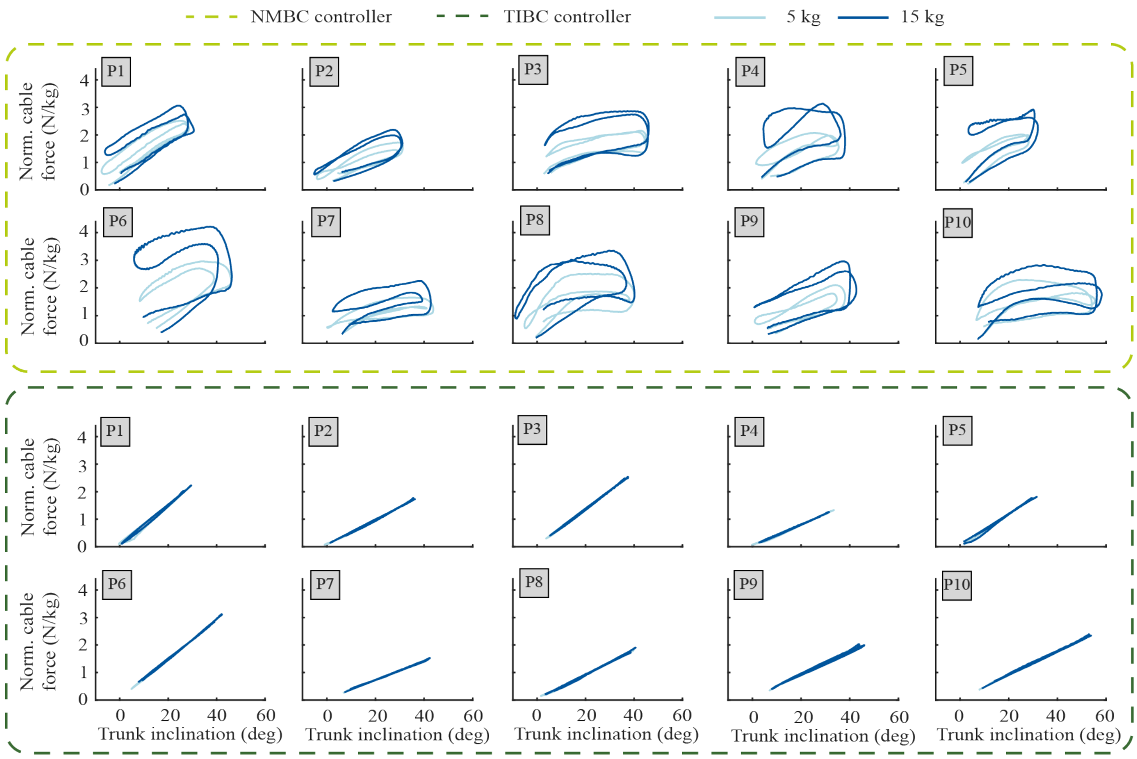

Figure C1. Subject-specific (for n = 10 participants) human-exosuit work loops during assisted box-lifting tasks, for neuromechanical model-based (NMBC) and trunk inclination-based (TIBC) controllers. Cable forces (summation of left and right cable and normalized to body weight) are plotted versus trunk inclination for 5 (light blue) and 15 kg (dark blue) conditions. P1 to P10 indicate the participant number.

D: Peak EMG during the complete cycle

Biomechanical metrics such as peak muscle activity and peak compressive forces are commonly studied to estimate the influence of cumulative damage over time (Gallagher et al., 2017). Here, we complement the results in the main text with comparison of differences in peak muscle activity across the three conditions (NOEXO, NMBC, and TIBC), as well as the two weights lifted (5 kg and 15 kg). Repeated-measures ANOVA with post-hoc correction was performed to estimate the significant differences across these conditions.

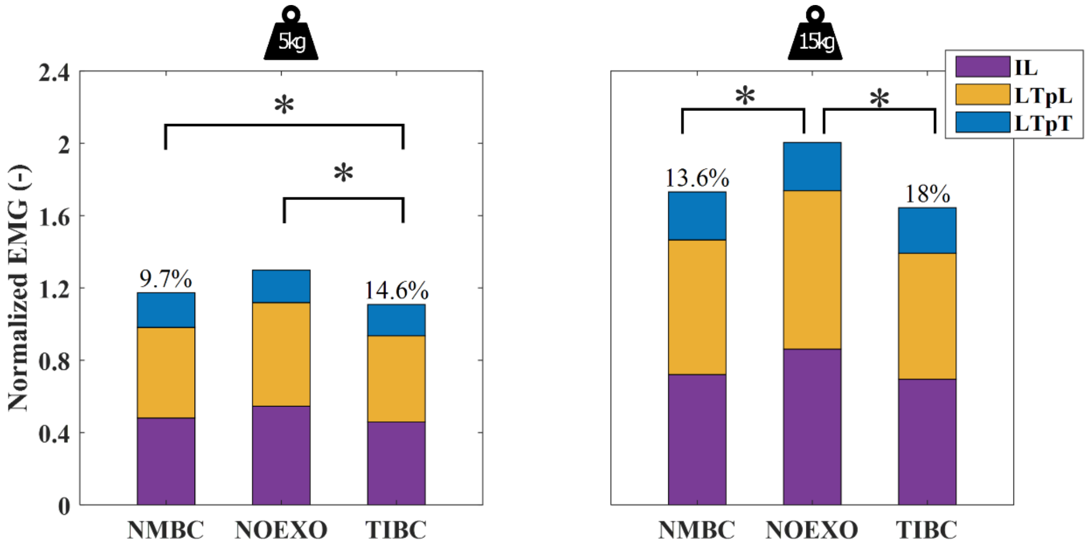

*Figure D1: Peak EMG values (averaged across the complete lifting cycle and participants) for iliocostalis (IL), longissimus thoracis pars lumborum (LTpL) and pars thoracis (LTpT), for NOEXO, neuromechanical model-based control (NMBC) and trunk inclination-based control (TIBC) conditions. The bars consist of blocks which depict the summation of left and right muscles. Numerical values indicate overall percentage of EMG reduction with respect to NOEXO condition. Statistically significant differences are denoted by * (p<0.05).*

Figure D1 shows the differences in peak muscle activity summed across the left and right side. We see that TIBC significantly reduced muscle activity compared to NOEXO condition for both weights lifted. For 5 kg condition, the TIBC reduced muscle activity compared to the NMBC condition. For 15 kg condition, the NMBC reduced muscle activity compared to NOEXO.

E: Peak EMG during parts of the lifting cycle

Here, we present the differences in peak muscle activity for the different phases of the lifting cycle. This includes the erect stance (40% to 60% of the cycle), lifting phase (25% to 40% and 75% to 100% of the cycle) and the lowering phases (1% to 25% and 60% to 75% of the cycle).

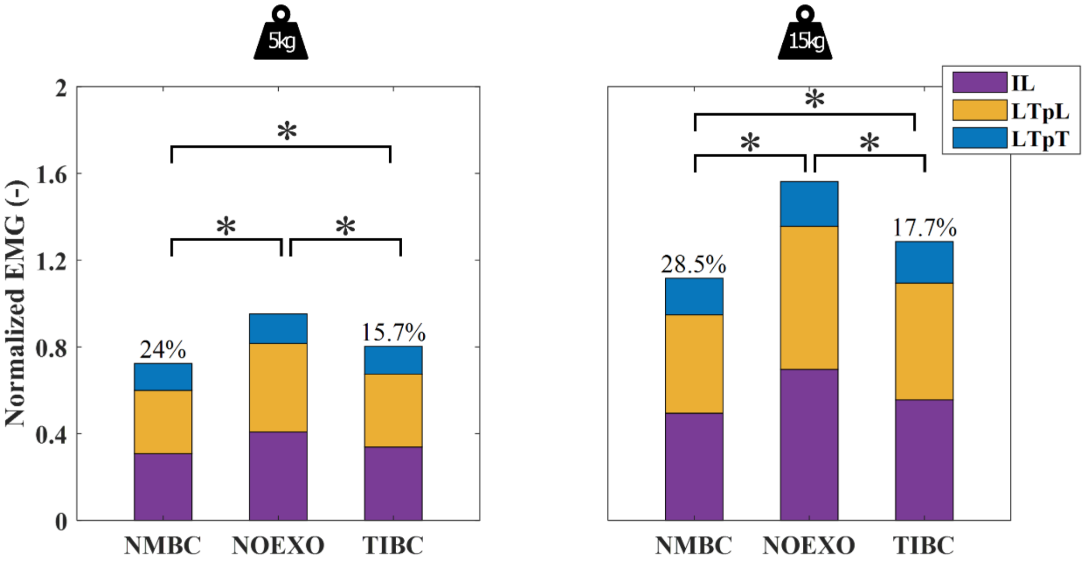

*Figure E1: Erect Stance: Peak EMG values for iliocostalis (IL), longissimus thoracis pars lumborum (LTpL) and pars thoracis (LTpT), for NOEXO, neuromechanical model-based control (NMBC) and trunk inclination-based control (TIBC) conditions. The bars consist of blocks which depict the summation of left and right muscles. Numerical values indicate overall percentage of EMG reduction with respect to NOEXO condition. Statistically significant differences are denoted by * (p<0.05).*

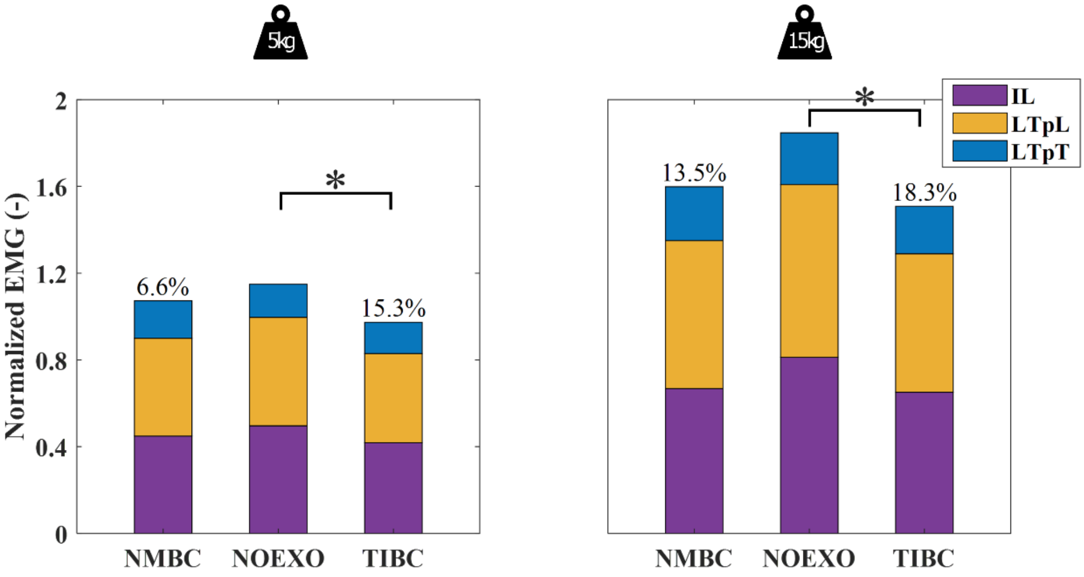

*Figure E2: Lifting Phase: Peak EMG values for iliocostalis (IL), longissimus thoracis pars lumborum (LTpL) and pars thoracis (LTpT), for NOEXO, neuromechanical model-based control (NMBC) and trunk inclination-based control (TIBC) conditions. The bars consist of blocks which depict the summation of left and right muscles. Numerical values indicate overall percentage of EMG reduction with respect to NOEXO condition. Statistically significant differences are denoted by * (p<0.05).*

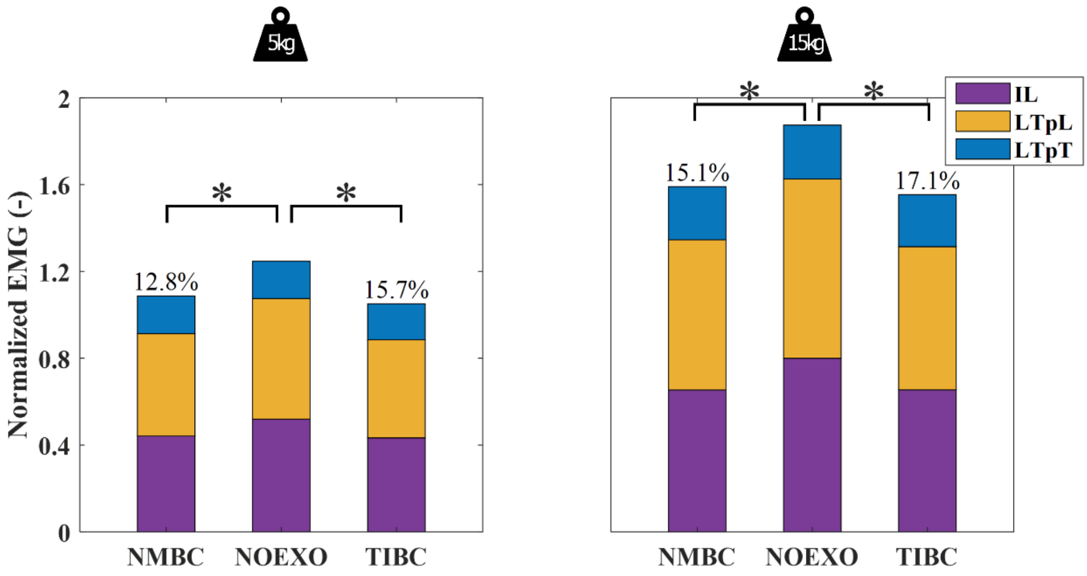

*Figure E3: Lowering Phase: Peak EMG values for iliocostalis (IL), longissimus thoracis pars lumborum (LTpL) and pars thoracis (LTpT), for NOEXO, neuromechanical model-based control (NMBC) and trunk inclination-based control (TIBC) conditions. The bars consist of blocks which depict the summation of left and right muscles. Numerical values indicate overall percentage of EMG reduction with respect to NOEXO condition. Statistically significant differences are denoted by * ($p<0.05$).*

Figure E1 shows that during erect stance, significant differences were found for peak muscle activity between NOEXO and both NMBC as well as TIBC. This was true for both weights lifted. Similarly, for both weight conditions, the NMBC reduced the peak activity more than TIBC. Figure E2 shows the differences in peak muscle activity only during the lifting phase of the tasks. Significant reductions were found by TIBC for both 5 and 15 kg. Finally, Figure E3 shows that during the lowering phase, both NMBC and TIBC showed reductions in muscle activity compared to the NOEXO condition. This was true for both weight conditions.

F: Peak L5/S1 Moments, and Compressive Forces during parts of the lifting cycle

Here, we present the differences in peak lumbosacral moments and compressive forces for the different phases of the lifting cycle. This includes the erect stance (40% to 60% of the cycle), lifting phase (25% to 40% and 75% to 100% of the cycle) and the lowering phases (1% to 25% and 60% to 75% of the cycle).

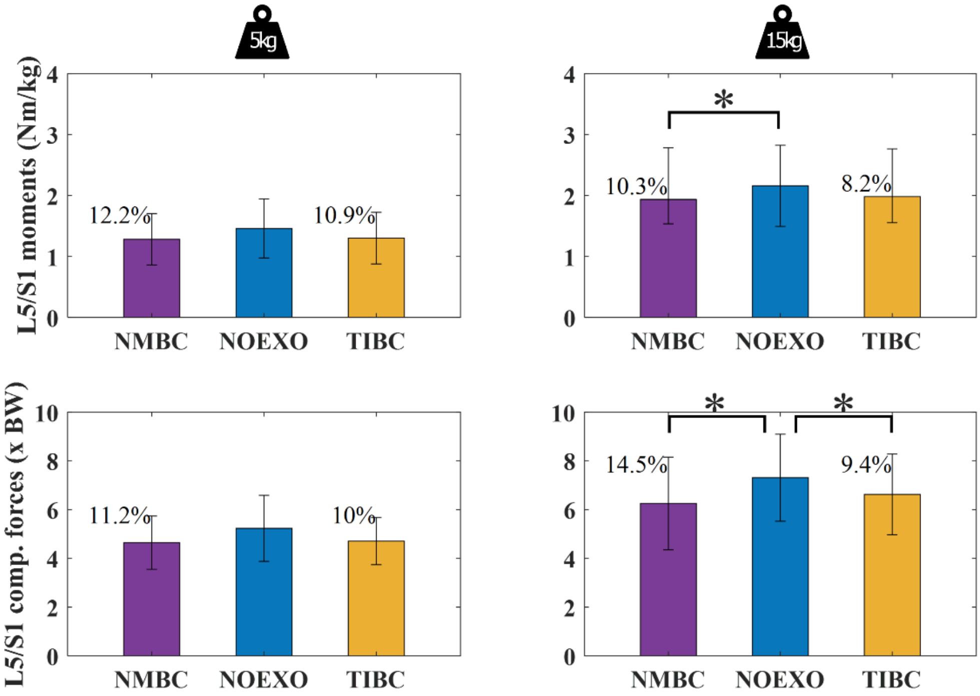

*Figure F1: Erect Stance: Peak L5/S1 flexion joint moments and compression forces for 5 and 15kg weight conditions, and NOEXO, neuromechanical model-based control (NMBC) and trunk inclination-based control (TIBC) conditions. Numerical values indicate overall moment or compression force reduction with respect to NOEXO condition. Statistically significant differences are denoted by * (p<0.05).*

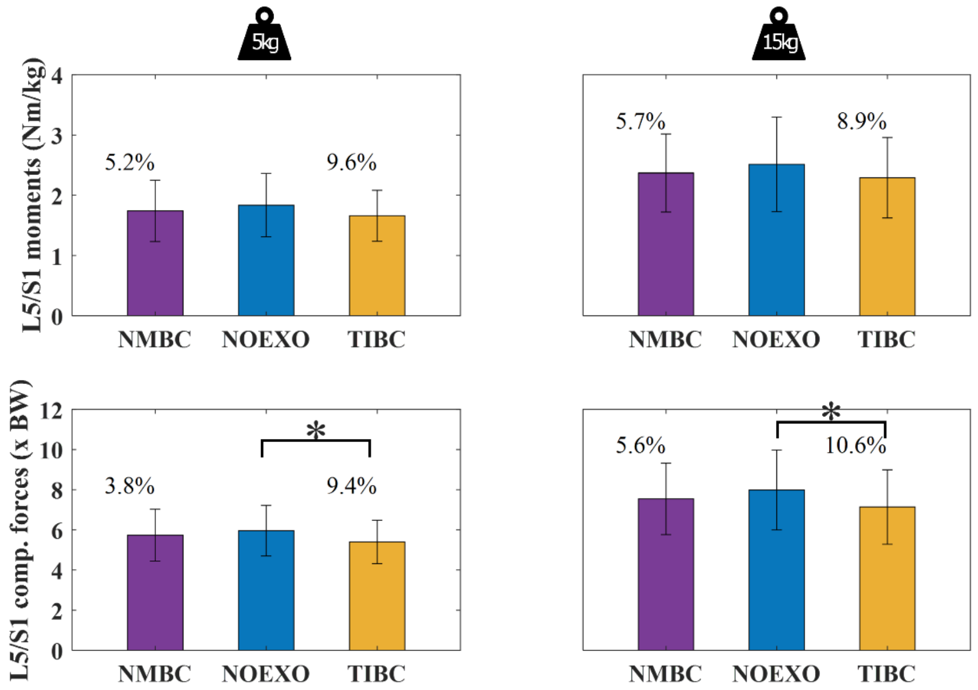

*Figure F2: Lifting Phase: Peak L5/S1 flexion joint moments and compression forces for 5 and 15kg weight conditions, and NOEXO, neuromechanical model-based control (NMBC) and trunk inclination-based control (TIBC) conditions. Numerical values indicate overall moment or compression force reduction with respect to NOEXO condition. Statistically significant differences are denoted by * (p<0.05).*

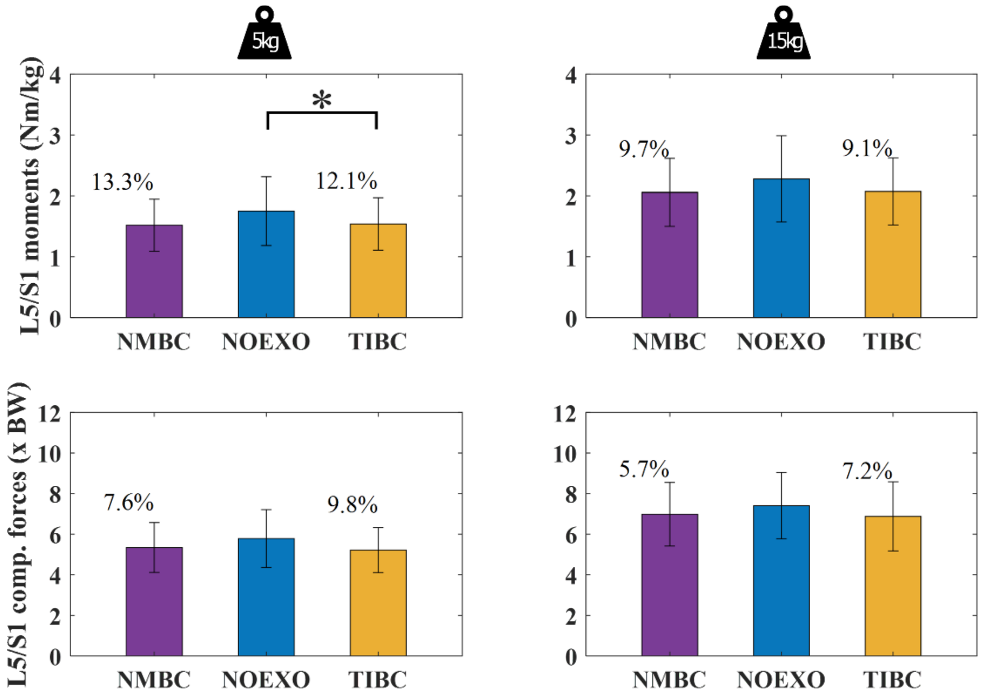

*Figure F3: Lowering Phase: Peak L5/S1 flexion joint moments and compression forces for 5 and 15kg weight conditions, and NOEXO, neuromechanical model-based control (NMBC) and trunk inclination-based control (TIBC) conditions. Numerical values indicate overall moment or compression force reduction with respect to NOEXO condition. Statistically significant differences are denoted by * (p<0.05).*

Figure F1 shows the differences in peak moments and compressive forces only during the erect stance. We see that NMBC reduces the peak moments as well as compressive forces compared to NOEXO condition for 15 kg weight. The TIBC only reduced the peak compressive forces when carrying the 15 kg weight. Figure F2 shows the differences during the lifting phase. Only peak compressive forces were reduced by the TIBC compared to the NOEXO condition, irrespective of the weight carried. Finally, figure F3 shows the differences during the lowering phase. Only TIBC showed significant reductions in peak moments when lifting 5 kg compared to the NOEXO condition.

G: Mean L5/S1 Moments, and Compressive Forces during parts of the lifting cycle

Here, we present the differences in mean lumbosacral moments and compressive forces for the different phases of the lifting cycle. This includes the erect stance (40% to 60% of the cycle), lifting phase (25% to 40% and 75% to 100% of the cycle) and the lowering phases (1% to 25% and 60% to 75% of the cycle).

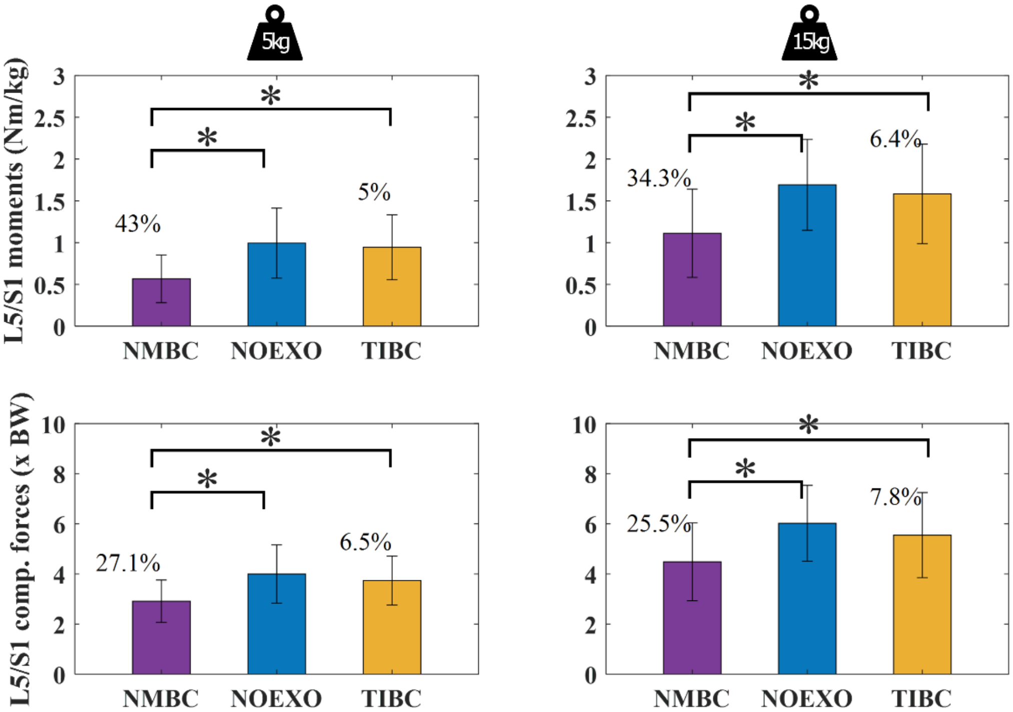

*Figure G1: Erect Stance: Mean L5/S1 flexion joint moments and compression forces for 5 and 15kg weight conditions, and NOEXO, neuromechanical model-based control (NMBC) and trunk inclination-based control (TIBC) conditions. Numerical values indicate overall moment or compression force reduction with respect to NOEXO condition. Statistically significant differences are denoted by * (p<0.05).*

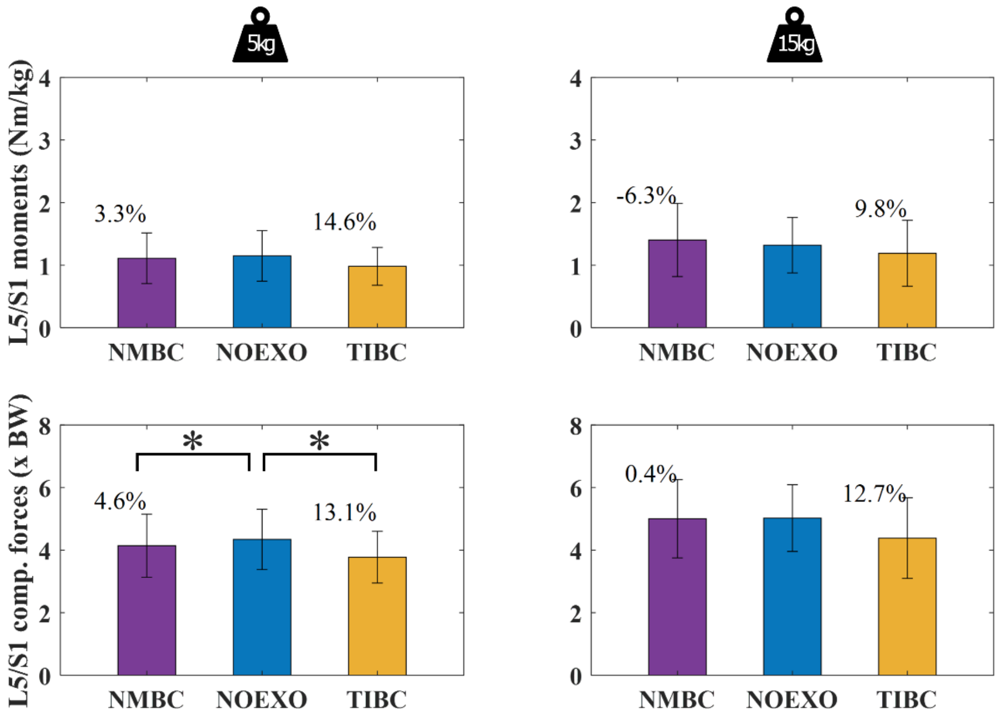

*Figure G2: Lifting Phase: Mean L5/S1 flexion joint moments and compression forces for 5 and 15kg weight conditions, and NOEXO, neuromechanical model-based control (NMBC) and trunk inclination-based control (TIBC) conditions. Numerical values indicate overall moment or compression force reduction with respect to NOEXO condition. Statistically significant differences are denoted by * (p<0.05).*

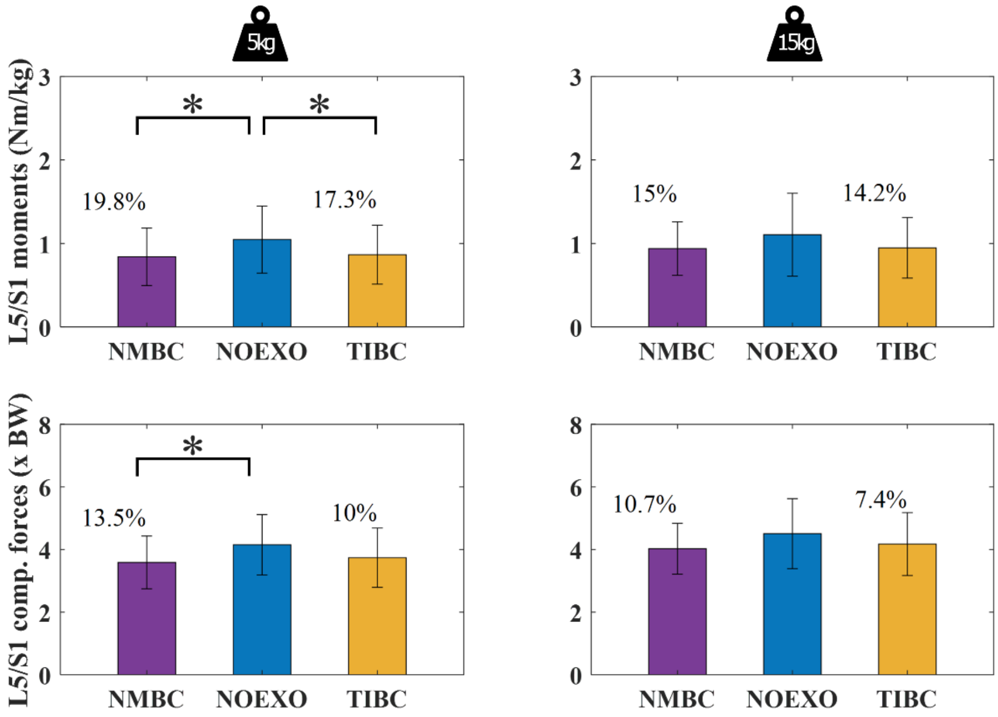

*Figure G3: Lowering Phase: Mean L5/S1 flexion joint moments and compression forces for 5 and 15kg weight conditions, and NOEXO, neuromechanical model-based control (NMBC) and trunk inclination-based control (TIBC) conditions. Numerical values indicate overall moment or compression force reduction with respect to NOEXO condition. Statistically significant differences are denoted by * (p<0.05).*

Figure G1 shows the differences in mean moments and compressive forces only during the erect stance. We see that irrespective of the weight carried, NMBC reduced both the mean moments and compressive forces compared to the NOEXO as well as TIBC. Figure G2 shows the differences during the lifting phase. Only mean compressive forces were reduced by the both NMBC and TIBC compared to NOEXO conditions when lifting 5 kg. Finally, figure G3 shows the differences during the lowering phase. Reductions were seen only for the 5 kg weights. NMBC reduced both mean moments and compressive forces, whereas, TIBC reduced the mean moments during the lowering phase.

REFERENCES

Chen, B., Lanotte, F., Grazi, L., Vitiello, N., & Crea, S. (2019). Classification of Lifting Techniques for Application of A Robotic Hip Exoskeleton. *Sensors*, *19*(4), 963. https://doi.org/10.3390/s19040963

Giovacchini, F., Vannetti, F., Fantozzi, M., Cempini, M., Cortese, M., Parri, A., Yan, T., Lefeber, D., & Vitiello, N. (2015). A light-weight active orthosis for hip movement assistance. *Robotics and Autonomous Systems*, *73*, 123–134. https://doi.org/10.1016/j.robot.2014.08.015

Govaerts, R., De Bock, S., Provyn, S., Vanderborght, B., Roelands, B., Meeusen, R., & De Pauw, K. (2024). The impact of an active and passive industrial back exoskeleton on functional performance. *Ergonomics*, *67*(5), 597–618. https://doi.org/10.1080/00140139.2023.2236817

Heo, U., Kim, S. J., & Kim, J. (2020). Backdrivable and Fully-Portable Pneumatic Back Support Exoskeleton for Lifting Assistance. *IEEE Robotics and Automation Letters*, *5*(2), 2047–2053. https://doi.org/10.1109/LRA.2020.2969169

Hiromasa Hara, & Yoshiyuki Sankai. (2010). Development of HAL for Lumbar Support. *SCIS & ISIS*.

Hyun, D. J., Lim, H., Park, S., & Nam, S. (2020). Singular Wire-Driven Series Elastic Actuation with Force Control for a Waist Assistive Exoskeleton, H-WEXv2. *IEEE/ASME Transactions on Mechatronics*, *25*(2), 1026–1035. https://doi.org/10.1109/TMECH.2020.2970448

Ko, H. K., Lee, S. W., Koo, D. H., Lee, I., & Hyun, D. J. (2018). Waist-assistive exoskeleton powered by a singular actuation mechanism for prevention of back-injury. *Robotics and Autonomous Systems*, *107*, 1–9. https://doi.org/10.1016/j.robot.2018.05.008

Koopman, A. S., Toxiri, S., Power, V., Kingma, I., van Dieën, J. H., Ortiz, J., & de Looze, M. P. (2019). The effect of control strategies for an active back-support exoskeleton on spine loading and kinematics during lifting. *Journal of Biomechanics*, *91*, 14–22. https://doi.org/10.1016/j.jbiomech.2019.04.044

Lanotte, F., McKinney, Z., Grazi, L., Chen, B., Crea, S., & Vitiello, N. (2021). Adaptive Control Method for Dynamic Synchronization of Wearable Robotic Assistance to Discrete Movements: Validation for Use Case of Lifting Tasks. *IEEE Transactions on Robotics*, *37*(6), 2193–2209. https://doi.org/10.1109/TRO.2021.3073836

Lee, J., & Kim, G. (2019). Design and Control of a Lifting Assist Device for Preventing Lower Back Injuries in Industrial Athletes. *International Journal of Precision Engineering and Manufacturing*, *20*(10), 1825–1838. https://doi.org/10.1007/s12541-019-00183-0

Miura, K., Kadone, H., Koda, M., Abe, T., Endo, H., Murakami, H., Doita, M., Kumagai, H., Nagashima, K., Fujii, K., Noguchi, H., Funayama, T., Kawamoto, H., Sankai, Y., & Yamazaki, M. (2018). The hybrid assisted limb (HAL) for Care Support, a motion assisting robot providing exoskeletal lumbar support, can potentially reduce lumbar load in repetitive snow-shoveling movements. *Journal of Clinical Neuroscience*, *49*, 83–86. https://doi.org/10.1016/j.jocn.2017.11.020

Schwartz, M., Desbrosses, K., Theurel, J., & Mornieux, G. (2022). Using passive or active back-support exoskeletons during a repetitive lifting task: influence on cardiorespiratory parameters. *European Journal of Applied Physiology*, *122*(12), 2575–2583. https://doi.org/10.1007/s00421-022-05034-x

Toxiri, S., Calanca, A., Ortiz, J., Fiorini, P., & Caldwell, D. G. (2018). A Parallel-Elastic Actuator for a Torque-Controlled Back-Support Exoskeleton. *IEEE Robotics and Automation Letters*, *3*(1), 492–499. https://doi.org/10.1109/LRA.2017.2768120

Toxiri, S., Koopman, A. S., Lazzaroni, M., Ortiz, J., Power, V., de Looze, M. P., O'Sullivan, L., & Caldwell, D. G. (2018). Rationale, Implementation and Evaluation of Assistive Strategies for an Active Back-Support Exoskeleton. *Frontiers in Robotics and AI*, *5*. https://doi.org/10.3389/frobt.2018.00053

Toxiri, S., Näf, M. B., Lazzaroni, M., Fernández, J., Sposito, M., Poliero, T., Monica, L., Anastasi, S., Caldwell, D. G., & Ortiz, J. (2019). Back-Support Exoskeletons for Occupational Use: An Overview of Technological Advances and Trends. *IISE Transactions on Occupational Ergonomics and Human Factors*, *7*(3–4), 237–249. https://doi.org/10.1080/24725838.2019.1626303

Walter, T., Stutzig, N., & Siebert, T. (2023). Active exoskeleton reduces erector spinae muscle activity during lifting. *Frontiers in Bioengineering and Biotechnology*, *11*. https://doi.org/10.3389/fbioe.2023.1143926

Wei, W., Zha, S., Xia, Y., Gu, J., & Lin, X. (2020). A Hip Active Assisted Exoskeleton That Assists the Semi-Squat Lifting. *Applied Sciences*, *10*(7), 2424. https://doi.org/10.3390/app10072424

Yamanaka, Y., Kashima, M., Arakawa, H., Nishihama, R., Yokoyama, K., & Nakamura, T. (2021). Verification of the "AB-Wear" Semi-Exoskeleton-Type Power-Assist Suit in Providing Assistance to the Lower Back. *2021 22nd IEEE International Conference on Industrial Technology (ICIT)*, 111–117. https://doi.org/10.1109/ICIT46573.2021.9453629

Yin, P., Yang, L., Wang, C., & Qu, S. (2019). Effects of wearable power assist device on low back fatigue during repetitive lifting tasks. *Clinical Biomechanics*, *70*, 59–65. https://doi.org/10.1016/j.clinbiomech.2019.07.023

Zhang, T., & Huang, H. H. (2018). A Lower-Back Robotic Exoskeleton: Industrial Handling Augmentation Used to Provide Spinal Support. *IEEE Robotics and Automation Magazine*, *25*(2), 95–106. https://doi.org/10.1109/MRA.2018.2815083
